# Preparation of Chitosan/Poly(Vinyl Alcohol) Nanocomposite Films Incorporated with Oxidized Carbon Nano-Onions (Multi-Layer Fullerenes) for Tissue-Engineering Applications

**DOI:** 10.3390/biom9110684

**Published:** 2019-11-01

**Authors:** Carlos David Grande Tovar, Jorge Iván Castro, Carlos Humberto Valencia, Diana Paola Navia Porras, José Herminsul Mina Hernandez, Mayra Eliana Valencia, José Daniel Velásquez, Manuel N. Chaur

**Affiliations:** 1 Grupo de Investigación de fotoquímica y fotobiología, Universidad del Atlántico, Carrera 30 Número 8-49, Puerto Colombia 081008, Colombia; carlosgrande@mail.uniatlantico.edu.co; 2 Grupo de Investigación SIMERQO, Departamento de Química, Universidad del Valle, Calle 13 No. 100-00, Cali 76001, Colombia; jorge.castro@correounivalle.edu.co (J.I.C.); jose.velasquez.carmona@correounivalle.edu.co (J.D.V.); 3 Escuela de Odontología, Grupo biomateriales dentales, Universidad del Valle, Calle 13 No. 100-00, Cali 76001, Colombia; carlos.humberto.valencia@correounivalle.edu.co; 4 Grupo de Investigación Biotecnología, Facultad de Ingeniería, Universidad de San Buenaventura Cali, Carrera 122 # 6-65, Cali 76001, Colombia; dpnavia@usbcali.edu.co; 5 Escuela de Ingeniería de Materiales, Facultad de Ingeniería, Universidad del Valle, Calle 13 No. 100-00, Santiago de Cali 760032, Colombia; valencia.mayra@correounivalle.edu.co; 6 Centro de Excelencia en Nuevos Materiales (CENM), Universidad del Valle, Calle 13 No. 100-00, Santiago de Cali 760032, Colombia

**Keywords:** biodegradable films, chitosan, oxidized carbon nano-onions, poly(vinyl alcohol), tissue engineering

## Abstract

Recently, tissue engineering became a very important medical alternative in patients who need to regenerate damaged or lost tissues through the use of scaffolds that support cell adhesion and proliferation. Carbon nanomaterials (carbon nanotubes, fullerenes, multi-wall fullerenes, and graphene) became a very important alternative to reinforce the mechanical, thermal, and antimicrobial properties of several biopolymers. In this work, five different formulations of chitosan/poly(vinyl alcohol)/oxidized carbon nano-onions (CS/PVA/ox-CNO) were used to prepare biodegradable scaffolds with potential biomedical applications. Film characterization consisted of Fourier-transform infrared (FTIR) spectroscopy, thermogravimetric analysis (TGA), differential scanning calorimetry (DSC), tension strength, Young’s modulus, X-ray diffraction spectroscopy (XRD), scanning electron microscopy (SEM), and energy-dispersive spectroscopy (EDS). The degradation in a simulated body fluid (FBS) demonstrated that all the formulations lost between 75% and 80% of their weight after 15 days of treatment, but the degradation decreased with the ox-CNO content. In vivo tests after 90 days of subdermal implantation of the nanocomposite films in Wistar rats’ tissue demonstrated good biocompatibility without allergenic reactions or pus formation. There was a good correlation between FBS hydrolytic degradation and degradation in vivo for all the samples, since the ox-CNO content increased the stability of the material. All these results indicate the potential of the CS/PVA/ox-CNO nanocomposite films in tissue engineering, especially for long-term applications.

## 1. Introduction

Designed scaffolds should be biocompatible and biodegradable, capable of producing an extracellular matrix (ECM) by supporting the cell’s adhesion and proliferation. They should also have porosity to promote nutrient transportation, and their mechanical properties should be similar to native tissue [1,2,3]. To meet these requirements, a high surface area and an interconnected three-dimensional (3D) porous structure with sizes ranging between 200 and 600 μm are essential, while also providing nutrient transfer, waste disposal, cellular infiltration, neovascularization, and cell proliferation [4,5,6].

Natural polymers are preferred materials for producing scaffolds, due to their better overall interactions with various cell types, and their lack of toxicity [7]. However, synthetic polymers are cheaper and allow chemical functionalization to assist cell adhesion despite the immune response [8]. The preferred synthetic polymers used for scaffold synthesis include poly(l-lactic acid) (PLLA), poly(glycolic acid) (PGA), poly(caprolactone) (PCL), and poly(lactic-*co*-glycolic) acid (PLGA) [9,10,11]. Composites with natural polymers are also available with better hydrophilicity, cell attachment, and biodegradability [12,13]. The most used biopolymers are chitosan, alginate (Alg), collagen (Col), gelatin (Gn), silk fibroin (SF), and glycosaminoglycans (GAGs) [13,14,15,16,17,18,19].

Chitosan (CS), a natural polymer obtained from the partial deacetylation of chitin, is one of the most common biopolymers on earth [20]. It is present in the shells of crustaceans such as crab and shrimp [21]. CS has many desirable properties for tissue engineering such as biocompatibility, non-toxicity, and biodegradability [22]. CS was used in several tissue-engineering applications involving bone [23,24], cartilage [25,26], liver [27], tendons [28,29], ligaments and nerves [30,31], wound healing [32,33], separation membranes [34,35,36], blood anticoagulants [37,38,39,40], contact lenses [41], controlled release of drugs [42,43,44], fat-sequestering agent [45,46], hydrogel preparations [47,48], and food packaging material [49,50]. CS was evaluated for bone tissue engineering (BTE) with good cell adhesion and proliferation observed [51,52,53,54]. CS composites with different materials such as ceramics, nanofillers, and polymers were evaluated for BTE with excellent biocompatibility and cell proliferation results [55,56,57,58,59,60,61]. It was recognized that amine and hydroxyl groups facilitate the addition of side groups for cell incorporation and recognition in BTE [62]. However, like many other polysaccharides, it presents some disadvantages such as poor mechanical properties, poor solubility in conventional solvents, and poor stability in physiological media due to the high amount of hydrogen bonding [63].

Many studies addressed the physicochemical attributes of nanocomposite polymers thanks to their effectiveness in overcoming the disadvantages of naturally occurring polymers [63,64]. Our group investigated the use of nanocomposites based on graphene oxide and chitosan for applications in tissue engineering [65,66,67,68,69,70,71]. Even with the nanofillers, there is still a need to achieve better mechanical and thermal stability combined with higher biocompatibility in nanocomposites of chitosan and carbon nanomaterials.

Carbon nano-onions (CNOs) are multi-shell fullerenes of 5 to 10 nm, discovered by Ugarte in 1992 upon impacting carbon nanostructures with an electron beam through a TEM experiment [72]. For the chemical derivatization of CNOs, various synthetic routes were applied, introducing new functional groups that increase their application possibilities. Some potential uses include optical applications, catalysis, and gas storage. CNOs are also useful in photovoltaic and fuel cell applications. They demonstrated a superior lubrication ability compared to conventional lubricants [73].

However, there are few reports of CS and CNO composites in the literature. The electrochemical properties of composites consisting of CNOs and poly(diallyl dimethylammonium chloride) (PDDA) or chitosan (CS) were reported [74,75]. The lack of literature regarding nanocomposites of CS and oxidized (ox-)CNOs for biomedical applications is evident. Very few studies were carried out to determine the cytotoxicity of CNOs. They were even used for the treatment of cancer [76]; however, many more studies are still required to show whether they are toxic or if, on the contrary, they promote cell adhesion and proliferation, while facilitating the recognition and permanence of scaffolds in animal tissues without immune responses [77]. There is a lack of information on their characterization including the physical, chemical, and mechanical evaluation of nanocomposite films based on CS/poly(vinyl alcohol) (PVA)/ox-CNO, together with biological tests in vitro and in vivo. The results of this investigation will allow collecting toxicological information for these nanocomposites, as well as evaluating their possible application in tissue engineering. Therefore, this research investigates nanocomposite films of PVA/CS reinforced with ox-CNOs for biocompatibility studies at 90 days of subdermal implantation in biomodel skin.

## 2. Materials and Methods

### 2.1. Materials

For pristine (p-)CNO synthesis, carbon nanodiamonds (Sigma-Aldrich, Palo Alto, CA, USA) of 5 nm average diameter were annealed in an oven (Nabertherm LHT 02/18, Lilienthal, Bremen, Germany) at 1600 °C for 2 h in an inert atmosphere of nitrogen; thereafter, amorphous carbon was eliminated by heating at 400 °C in air for 2 h [78]. For the synthesis of ox-CNO, concentrated sulfuric acid (H_2_SO_4_), concentrated nitric acid (HNO_3_), and sodium hydroxide (NaOH) supplied by Merck (Burlington, MA, USA) were used. Nanocomposite films were produced using chitosan of low molecular weight (molecular weight (Mw.) 144,000 g/mol, deacetylation degree 89–90%), poly(vinyl alcohol) (PVA) (hydrolysis degree 87–89%, Mw. 93,000 g/mol, Sigma-Aldrich, Palo Alto, CA, USA). Glacial acetic acid from Merck (Burlington, MA, USA) was used for the preparation of CS solutions. Simulated biological fluid (FBS) was prepared using NaCl, K_2_HPO_4_·3H_2_O, CaCl_2_, Na_2_SO_4_, and tris-(hydroxymethyl aminomethane) ((CH_2_OH)_3_CNH_2_) acquired from Sigma Aldrich (Palo Alto, CA, USA), a well as NaHCO_3_, KCl, and MgCl_2_·6H_2_O from Fisher Chemical (Pittsburgh, PA, USA), and hydrochloric acid (HCl) from Merck (Burlington, MA, USA). All reagents were analytical grade.

### 2.2. Methods

#### 2.2.1. p-CNO Synthesis and ox-CNO

**Scheme 1:**
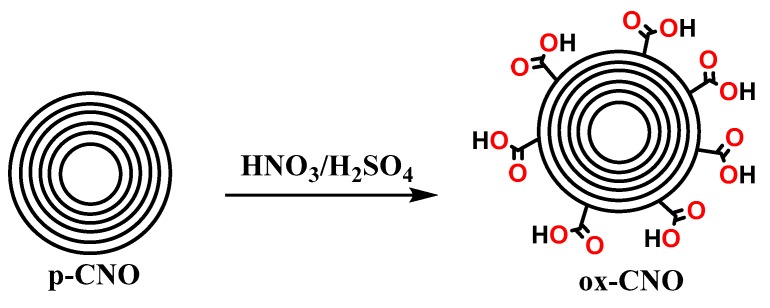
Oxidation of pristine carbon nano-onions (p-CNOs) [79].

For p-CNO synthesis, it was necessary to anneal ultra-dispersed carbon nanodiamonds of 5 nm average diameter in an oven at 1600 °C for 2 h in a nitrogen atmosphere. Then, amorphous carbon was eliminated by heating at 400 °C in air for 2 h. For the synthesis of ox-CNO (Scheme 1), a mixture of HNO_3_:H_2_SO_4_ (3:1) (20 mL) and 200 mg of p-CNO under reflux for 5 h was prepared. The resulting mixture was separated by means of centrifugation and subsequently neutralized with a solution of 1 M NaOH. Finally, the impurities were removed by washing with water, methanol, and ethanol, and then the sample was vacuum-dried at 60 °C for 12 h [79].

#### 2.2.2. Characterization of ox-CNOs

The ox-CNOs were chemically characterized by Fourier-transform infrared spectroscopy (FT-IR-8400) on an IR Affinity-1 infrared spectrophotometer (Shimadzu, Kyoto, Japan) in a range of 500–4000 cm^−1^ in transmittance mode using the diamond tip method.

The ox-CNOs and the p-CNOs were also analyzed through a ThermoScientific X-ray diffraction (XRD) (Thermo Scientific, Waltham, MA, USA) Smart Raman laser beam 532 nm.

The characteristic bands of the X-ray spectrum of p-CNO and ox-CNO were taken on a PANalytical X’Pert PRO diffractometer (Malvern PANalytical, Jarman Way, Royston, UK) using Cu Kα1 radiation (1.540598 Å) and Kα2 (1.544426 Å), with an electron accelerator voltage of 45 kV, an electron-generating current of 40 mA, an optical grid of incident beam 1°, and a diffracted beam grid of 9.1 mm, in a range 2θ between 5° and 40°. We used the Bragg law (Equation (1)) and the Scherrer (Equation (2)) equation to determine the average crystal size and the interlayer distance of the carbon nano-onions.

(1) $$d=\frac{\lambda }{2sen\theta }.$$

(2) $$\tau =\frac{K\lambda }{\beta cos\theta }.$$

#### 2.2.3. Nanocomposite Film Preparation

Nanocomposite films were prepared according to the previously reported methodology [67,70], using the formulations described in Table 1. For the preparation of the solutions, PVA and CS were dissolved in a 2% (*v*/*v*) acetic acid solution in different containers to reach a final concentration of 2% (wt.%) according to the wt.% ratio of each formulation. After that, a dispersion of ox-CNO (10 mg/mL) in 2% acetic acid was prepared using an ultrasonic bath (Branson, Madrid, Spain) for 2 h. Finally, the components were mixed homogeneously.

The homogeneous solutions were added to acetate molds, subsequently curing for 24 h in air, before being placed for 24 h in an oven at a temperature of 40 ± 0.2 °C, thereby obtaining a solid film of CS/PVA/ox-CNO. Once collected, the test specimens were cut and conditioned for the tensile test according to ASTM D6287, ASTM D618, and ASTM D882. Thicknesses were determined using a Mitutoyo digital micrometer No. 293-330 (Kawasaki, Japan), with three average values from each sample. The samples were placed in a desiccator at 10% relative humidity (RH) until the time of the test.

#### 2.2.4. Film Characterization

##### Fourier-Transform Infrared Spectroscopy (FTIR)

Functional groups of the films were identified using FTIR in ATR mode (attenuated total reflectance) with a diamond tip (Shimadzu, Kyoto, Japan).

##### X-ray Diffraction (DRX)

The characteristic bands of an X-ray spectrum of the films were determined using the same equipment and the same parameters and equations described before.

##### Scanning Electron Microscopy (SEM)

The nanocomposite film surface morphology was analyzed using a scanning electron microscope (SEM) (JEOL JSM-6490LA, Musashino, Tokyo, Japan). The voltage used was 20 kV with the mode of secondary backscattered electrons. For the proper conductivity of the samples, a coating of gold was prepared.

##### Tensile Strength of Films

For the tensile test, a universal SHIMADZU EZ-LZ test machine (Shimadzu, Kyoto, Japan) was used, following the ASTM D882 standard. At least six samples per formulation were used. The gap between jaws was 100 mm, the width of the film was 20 mm, and the test speed was 50 mm/min.

##### Thermal Studies

Thermal properties of the polymers were measured by thermal gravimetric analysis (TGA) on a TA Instruments TGA Q50 V20.13 Build 39 (TA instrument, Delaware, New Castle, USA). The samples were heated up to 1000 °C at a heating rate of 10 °C/min under air atmosphere (flow rate 80 mL/min). The glass transition temperature (T_g_) was determined by differential scanning calorimetry using a DSC2A-00181 (TA instrument, Delaware, New Castle, USA) from the midpoint of the inflection tangent from the second heating at 10 °C/min. TGA and DSC data were analyzed using TA Instruments’ Universal Analysis software.

##### Degradation in Simulated Biological Fluid

The hydrolytic degradation was carried out according to the previously reported procedure [67] based on the ASTM F1635-16 standard. Nanocomposite films were immersed in FBS (prepared as previously reported) at 37 °C for 15 days in a Memmert IN 110 incubator (Memmert, Schwabach, Germany) [67]. The hydrolytic degradation was established on days one, four, seven, 11, and 15 of immersion. The weight loss (% $W_{l}$) was calculated according to Equation (3).

(3) $$W_{l}\;\left(\%\right)=\frac{W_{0}-W_{d}}{W_{0}}\;\times 100.$$

The initial weight of the samples prior to immersion was $W_{0}$, and the weight after drying for 48 h in the incubator at 37 °C was recorded as $W_{d}$.

The pH of the FBS was measured every day until the total test time was completed using an Accumet™ AB150 pH meter (Fisherbrand, Ottawa, ON, Canada).

After 15 days, the samples were adhered on carbon tape, and then a superficial gold coating was performed (Denton Vacum Model Desk IV equipment) to generate a conductive surface. Subsequently, the JEOL Model JSM 6490 LV microscope was used inspect the samples in the secondary electron mode with an acceleration voltage of 20 kV to obtain electron microscope images. Additionally, chemical microanalysis was carried out on several inspection areas, employing the energy-dispersive spectroscopy (EDS) probe of the Oxford Instrument Model INCAPentaFETx3 (Abingdon, UK). The EDS probe had a resolution of 137 eV to 5.9 keV, while SEM had a resolution of 15 nm with an acceleration voltage of 1 kV and a working distance of 6 mm in the secondary electron mode.

##### Biomodel Tests In Vivo

The immune response of the nanocomposite films was studied using Wistar rat subcutaneous tissue implantation [67,69,70]. Nanocomposite films of10 mm in diameter and 2 mm in thickness were implanted in subdermal tissue according to ISO 10993-6. Commercial porcine collagen was used as a control. Wistar rats were supplied by the Bioterio of the Universidad del Valle. The procedures carried out were approved by the Animal Ethics Committee of Universidad del Valle, by the CEAS 001-016 certificate.

After 30, 60, and 90 days of implantation, the samples were recovered, fixed in buffered formalin, dehydrated in alcohol solutions of ascending concentration (70%, 80%, 95%, and 100%), diaphanized with xylol, and infiltrated with paraffin for later cutting to 4 µm. The samples were processed for histological analysis by hematoxylin and eosin (H&E) and Masson trichromacy (MT) techniques.

##### Statistical Analysis

The analysis of variance (ANOVA) and the Tukey method for mean separation, with a confidence level of 95% (α = 0.05), were used to evaluate the effect of the different formulations on the mechanical properties and hydrolytic degradation. In vivo studies, mechanical properties, and hydrolytic degradation are presented as mean values of at least three replicates ± SD. The Statgraphics Centurion XVI program was used for these statistical analyses.

## 3. Results and Discussion

### 3.1. ox-CNO Characterization

#### 3.1.1. Fourier-Transform Infrared Spectroscopy (FTIR)

Figure S1 (Supplementary Materials) shows the FTIR spectrum of pristine carbon nano-onions (p-CNOs) and oxidized carbon nano-onions (ox-CNOs). The broad band at 1730 cm^−1^ resulted from the C=O stretching vibrations of carboxyl and carbonyl groups. The band between 1540 and 1590 cm^−1^ was also related to carboxyl and carbonyl groups [80]. Also, bands around 3200–3675 cm^−1^ and 1640–1660 cm^−1^ related to adsorbed water were observed for p-CNO. It was previously noted that some traces of water could be adsorbed onto the surface of carbon nanotubes and this could also have been the reason for the presence of these bands in pristine carbon nano-onions [80]. However, there were some additional bands in the FTIR spectrum for ox-CNO that confirmed the oxidation process of CNOs after 5 h. The broad band between 2900 and 3300 cm^−1^ corresponded to the presence of many COOH groups. All these facts confirmed the oxidation of the pristine carbon nano-onions.

#### 3.1.2. X-ray Diffraction (DRX)

Powder X-ray diffraction (XRD) is a useful technique to analyze the average crystal size and the interlayer distance of the carbon nano-onions, using Equations (1) and (2), respectively.

The term λ in the Bragg law (Equation (1)) is the X-ray wavelength, d is the interlayer distance, and θ is the Bragg’s angle. Also, in the Scherrer equation (Equation (2)), λ is the X-ray wavelength, k is a dimensionless shape factor with a value of 0.9, and β is the full width at half maximum (FWHM) in radians.

Figure S2 (Supplementary Materials) shows the X-ray diffraction pattern of p-CNO and ox-CNO. The nanocomposites exhibited a broad peak at 43.5°, which corresponded to crystal planes with inter-planar distance (100), while a sharp peak appeared at 44.4°, corresponding to the reflection of the plane (111) that appertained to untransformed planes of traces of carbon nanodiamonds during the graphitization process [29,30,31,32,33]. Through Equation (2) and the peak (100) of the diffractogram, an average size of 13.65 nm was calculated. The average interplanar distance was determined from the position to the reflection broad intense of the plane (002) at 25.2°. Finally, the number of average layers of nanocomposites could be calculated as five shells using Equation (4).

(4) $$n=\frac{\tau }{d}.$$

#### 3.1.3. Raman Spectroscopy

**Figure 1:**
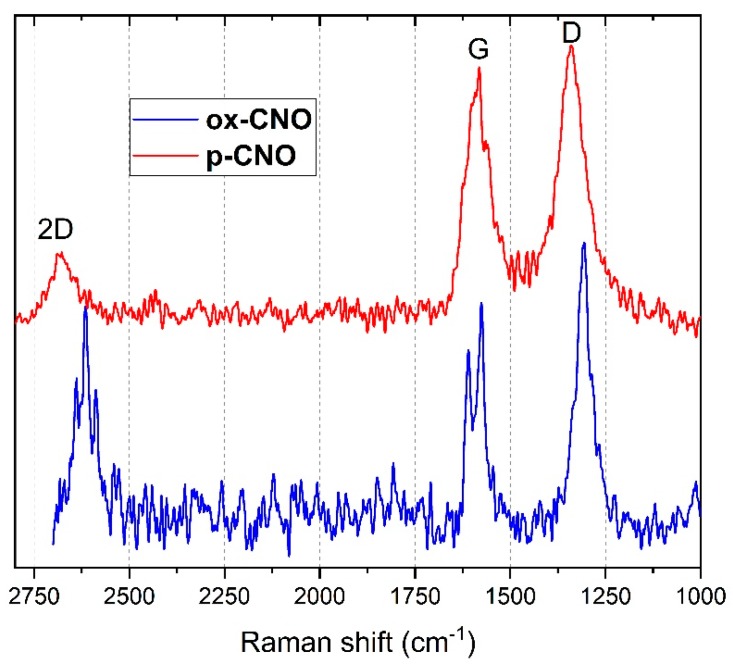
Raman spectrum of the oxidized carbon nano-onions (ox-CNOs) and pristine (p-)CNOs.

Raman spectroscopy is a very useful technique to characterize the typical graphitic structure of CNOs, which features two broad peaks in the area between 1300 and 1600 cm^−1^. Raman spectroscopy characterizes the material’s amount of order by analyzing the ratio of *sp*^2^ and *sp*^3^ carbon atoms, which is related to the intensity and the shape of the so-called D and G bands. According to the Raman spectrum of CNOs in Figure 1, the G band around 1580 cm^−1^ was attributed to the E_2g_ mode of *sp*^2^-hybridized carbons, and the D band was related to the breathing mode of the *sp*^2^ carbon rings, activated by defects in the fullerene network due to the presence of oxygen-rich functional groups, as well as to an increase in the *sp*^3^ hybridized carbons as a consequence of the increasing disorder by covalent functionalization [74]. The relationship between the intensities of bands D and G is directly related to the degree of oxidation [37]. The band at 2621 cm^−1^ was attributed to highly ordered graphitic materials. On the other hand, the ratio of intensities of the D and G bands (R = I_D_/I_G_) indicates the disorder density of the CNOs (due to the oxidation reaction in the outer shell). As previously reported, increases in R should be directly related to the oxidation degree of the CNOs [80]. R = I_D_/I_G_ for the pristine CNO was 1.07, and that for ox-CNO was 1.23, indicating that successful oxidation was obtained for the surface of CNOs.

#### 3.1.4. Thermogravimetric Analysis of ox-CNO

TGA is a useful technique to understand the disorder degree in carbon nanostructures and the degradation temperature of the different nanocomposite films. Through the thermogram of the ox-CNO, the degree of derivatization of the p-CNOs was calculated using the decomposition temperature at 514 °C. Usually, p-CNOs exhibit a decomposition temperature around 670–680 °C; however, when they are functionalized, the decomposition temperature decreases due to the introduction of “structural defects” at the surface (oxidation). Figure S3 (Supplementary Materials) shows two decomposition temperatures evidenced through the derivative TGA (DTGA), corresponding to the functionalization of carboxylic groups and carbonyl or hydroxyl groups at the CNO layer at 263 °C and 170 °C, respectively.

To calculate the average number of functional groups on the surface, we used the number of shells calculated through XRD. Using the result of five shells, the number of carbons in each shell was calculated using Equation (5) [81].

(5) $$60\;\times \;n^{2},$$

where n is the number of shells and 60 is the number of carbons of the core–shell (a fullerene). The outer shell contained 1500 carbon atoms, and the total number of carbon atoms was the addition of each shell, meaning that the skeleton CNO contained a total of 3300 carbons.

On the other hand, the weight loss of 10% at 283 °C corresponded to the carboxylic groups present on the surface of the CNO [81]. Therefore, the average mass of CNO could be obtained from the product of the total number of carbon atoms and the mass of CNO (3300 × 12 = 39,600 g/mol), corresponding to 90% of the compound (44,000 g/mol). On the other hand, the molar mass of the carboxylic functional group was 45 g/mol and the 10% loss should have corresponded to 4400 g/mol, which meant a presence of 98 functional groups, roughly one for every 15 (1500/98) carbons of the outer shell.

### 3.2. Film Characterization

#### 3.2.1. Fourier-Transform Infrared Spectroscopy (FTIR)

**Figure 2:**
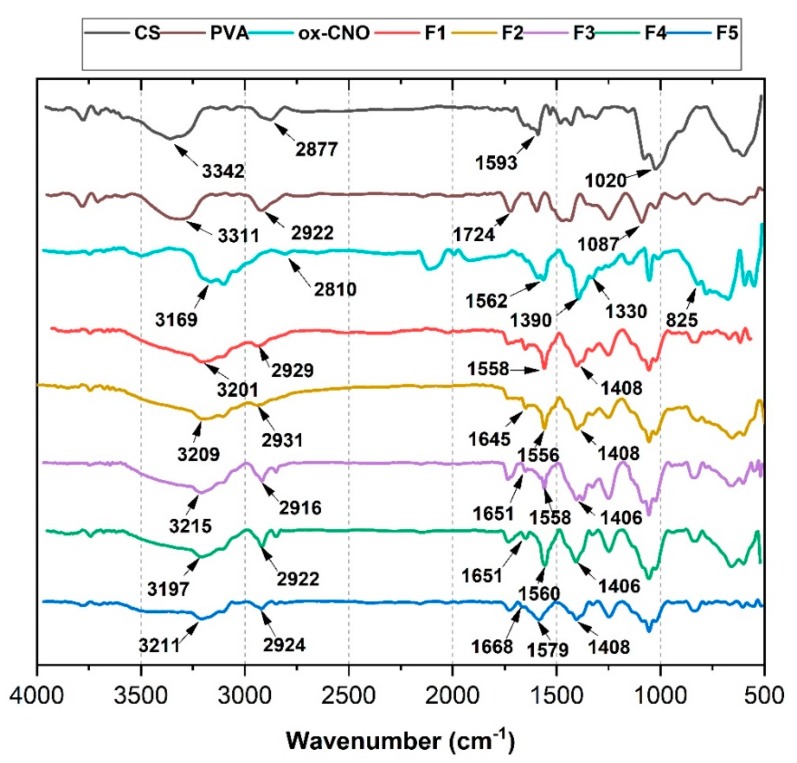
Attenuated total reflectance Fourier-transform infrared spectroscopy (ATR-FTIR) of chitosan (CS)/poly(vinyl alcohol) (PVA)/ox-CNO films. Formulations: F1 (CS:PVA:ox-CNO 30:70:0); F2 (CS:PVA:ox-CNO 29.75:70:0.25); F3 (CS:PVA:OX-CNO 29.50:70:0.50); F4 (CS:PVA:OX-CNO 29.25:70:0.75); F5 (CS:PVA:ox-CNO 29.00:70:1.00).

Figure 2 shows the FTIR spectrum of the composite films of CS/PVA/ox-CNO. It can be observed that F1 had the characteristic bands of PVA and CS [82]. The acetamido asymmetric stretching bands were observed at 1640 and 1560 cm^−1^. Nanocomposite films exhibited a shift to lower frequencies of the chitosan bands due to hydrogen bonding between PVA and the chitosan amine and hydroxyl groups. Between 3000 and 3500 cm^−1^, there was a broad band corresponding to both groups, with an evident shift due to the hydrogen bonding. CS/PVA composite films (F1) had absorption bands at 3291 and 2914 cm^−1^ related to –OH and –CH_2_ from PVA [83]. On the other hand, the ox-CNO spectrum displayed a C=O band of the carboxylic group and a C=C band at 1736 and 1618 cm^−1^, respectively [84].

The bands at 1647, 1563, and 1327 cm^−1^ were assigned to amides I and III of C=O stretching vibration, N–H bending of NH_2_, and CH_2_ wagging coupled with the OH group from CS. The peak at 1411 cm^−1^ corresponded to oscillations of OH and CH groups [85]. CS/PVA/ox-CNO composite film spectra presented a new band at 1750 cm^−1^ due to –COOH groups from ox-CNO. Broadening and shifting to a lower value of the –OH stretching vibration band from 3280 cm^−1^ to 2980 cm^−1^ could have been the result of the hydrogen-bonding interaction between ox-CNO and CS/PVA.

#### 3.2.2. X-Ray Diffraction (DRX)

**Figure 3:**
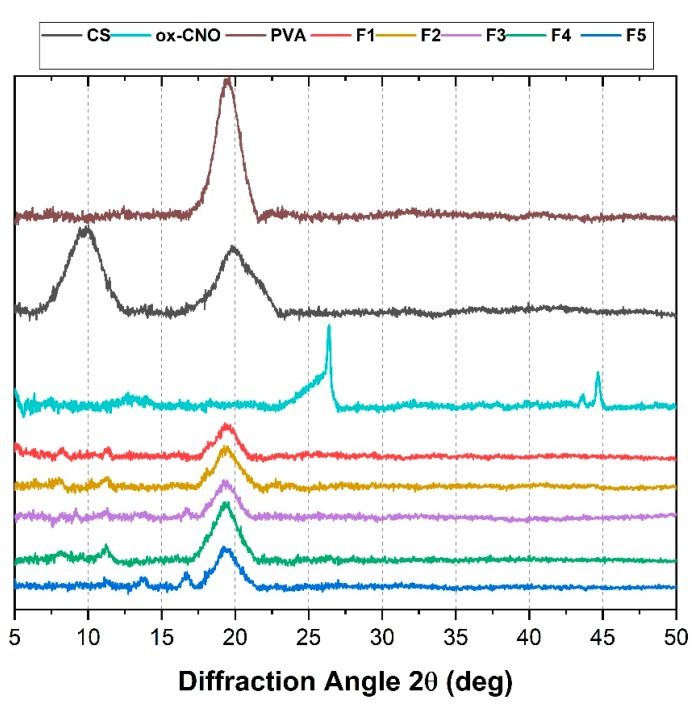
X-ray diffraction (XRD) of CS/PVA/ox-CNO films. Formulations: F1 (CS: PVA:ox-CNO 30:70:0); F2 (CS:PVA:ox-CNO 29.75:70:0.25); F3 (CS:PVA:ox-CNO 29.50:70:0.50); F4 (CS:PVA:ox-CNO 29.25:70:0.75); F5 (CS:PVA:ox-CNO 29.00:70:1.00).

The crystallinity of the CS/PVA/ox-CNO composite films was studied by XRD (Figure 3). Here, 2θ = 10.92° corresponded to a characteristic diffraction the ox-CNO peak attributed to a d spacing of 8.06° calculated from the Bragg equation [86]. For ox-CNO, peaks at 2θ = 26° and 44° were associated with the (002) and (100) diffractions of the hexagonal graphite structure [87]. Pure CS showed a peak at 2θ = 19.83° related to its crystalline structure [87]. The high number of hydrogen bonds in PVA produced a semi-crystalline structure with two characteristic peaks: One peak at 2θ = 19.8° with high intensity and one peak at about 2θ = 40.5° with a very low intensity [88]. The XRD pattern of the CS/PVA blend showed two very weak diffraction peaks located at 2θ = 8.03° and 11.3° assigned to CS, indicating an almost amorphous structure for this compound. The broad peak appearing at 2θ = 19.82° belonged to PVA crystallites [89]. Hydrogen bonds caused a shifting due to the crystallinity changes. In the CS/PVA/ox-CNO composite film spectra, it was observed that the incorporation of ox-CNO introduced three new peaks at 2θ = 11.03°, 12.93°, and 16.95°. Increasing the amount of ox-CNO in the nanocomposites affected the peak centered at 2θ = 19.8° corresponding to PVA. There were no characteristic peaks of ox-CNO, indicating a good dispersion within the CS/PVA matrix, which also indicated the low content of ox-CNO aggregates. The crystalline structure of CS/PVA composites was affected by the presence of carbon nanomaterials [90,91], as a result of the breaking of the hydrogen bonds of CS/PVA composites, which caused a decrease in crystallinity [92].

#### 3.2.3. Scanning Electron Microscopy (SEM)

**Figure 4:**
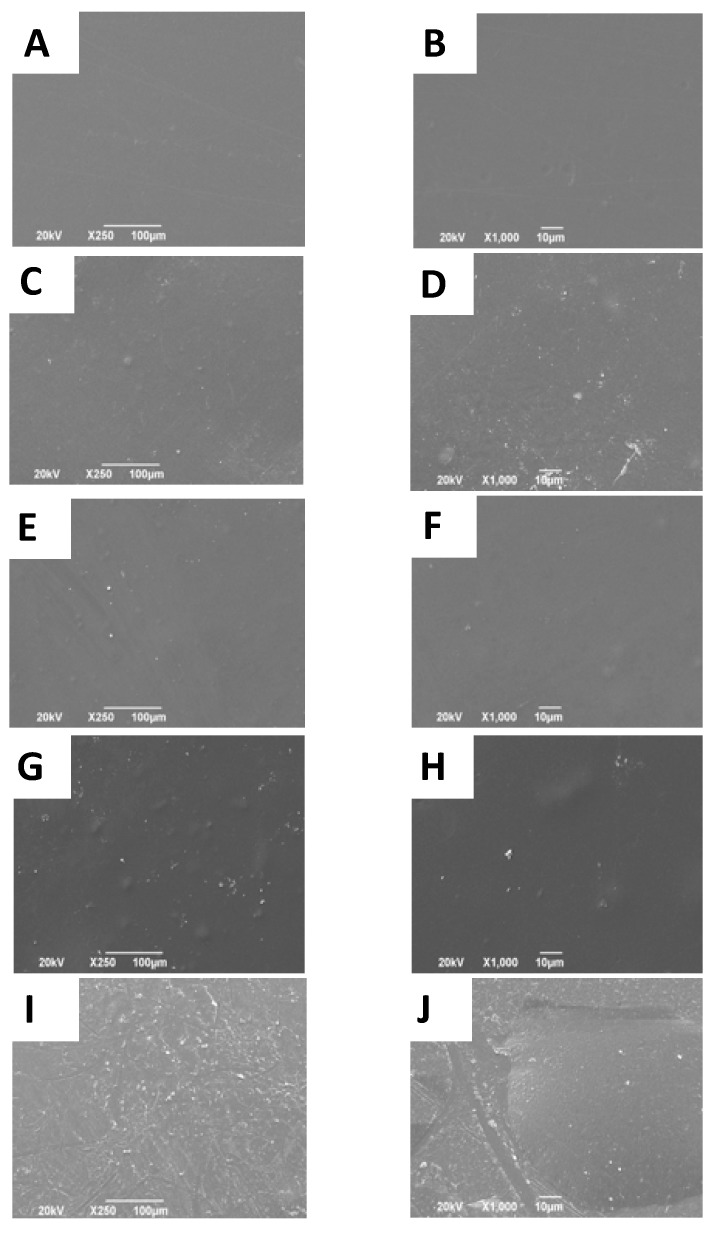
Morphology of the films: F1 (30 CS/70 PVA/0 ox-CNO) (**A**) at 250×, (**B**) at 1000×; F2 (29.75 CS/70 PVA/0.25 ox-CNO) (**C**) at 250×, (**D**) at 1000×; F3 (29.50 CS/70 PVA/0.50 ox-CNO) (**E**) at 250×, (**F**) at 1000×; F4 (29.25 CS/70 PVA/0.25 ox-CNO) (**G**) at 250×, (**H**) at 1000×; F5 (29.00 CS/70 PVA/1.00 ox-CNO), (**I**) at 250×, (**J**) at 1000×.

SEM images were used to analyze the surface morphology of the nanocomposite films. Figure 4 exhibits a typical SEM image of the surface of the CS/PVA/ox-CNO composite films. Figure 4A,B exhibit a smooth surface for the CS/PVA composite due to the semi-crystalline structure supported by the hydrogen bonding, whilst the morphology of the nanocomposite became rough and exhibited undulations due to ox-CNO presence. Previous studies showed that the surfaces of pure CS and PVA films are smooth, continuous, and compact [67], and because of the hydrogen bonding in the blend, the surface should also be smooth. By adding ox-CNO to the CS/PVA blend, the nanomaterial increased the roughness of the films due to their texture [83]. Some porosity could have occurred following rapid solvent evaporation, air bubble formation, or separation of polysaccharide molecules during the curing process of the films [93].

Films of CS/PVA blends were transparent as a result of the crystallinity of both polymers [83]. With increasing ox-CNO content, there was a darkening of the films and also an increase in opacity [94].

#### 3.2.4. The Tensile Strength of the Films

Materials for biomedical applications should have thermal and mechanical resistance with biocompatibility [83]. FTIR, XRD, and SEM analyses supported the hydrogen bonding between the ox-CNO and the CS/PVA composite, which also produced an increase in the roughness of the films. For this reason, the ox-CNO dispersed well in the polymer matrix and produced an improvement in the mechanical properties of the material with good thermal behavior.

The mechanical behavior of the different formulations was evaluated using Young’s modulus and the tensile strength. Figure S4 (Supplementary Materials) shows an improvement in the mechanical properties of the nanocomposites compared to CS/PVA (30/70) composites. The addition of 0.25% ox-CNO produced an increase of 0.83 MPa in tensile strength. However, Young’s modulus decreased by 0.93 GPa, as a consequence of the crystallinity lost (Table 2). An increase in the ox-CNO content caused a significant increase in the tensile modulus of the composite, e.g., loading 1.0 wt.% ox-CNO within the CS/PVA matrix generated increases of about 38% in Young’s modulus and 27% in tensile strength. This could be explained by the uniform distribution of the ox-CNO that caused its interaction with the polymer composite, modifying the original crystallinity of the material and also producing a more uniform distribution of the stress in the material, making it less concentric [90]. The incorporation of the nanocomposite allowed an increase in Young’s modulus and tensile strength. It is possible that, as the amount of the nanomaterial was increased, it could be better dispersed and, thus, the mechanical properties of the material improved. In the XRD analysis, a drastic change in the crystallinity of the material was not observed, which was evidenced in the mechanical properties, which generally improved with respect to the pure CS/PVA composite. Similar behavior was observed for carbon nanotubes [95] and GO composites [90], where it was shown that a large increase in mechanical properties requires a large increase in the crystallinity of the polymer. According to Figure S4 (Supplementary Materials), the tensile strength was affected by the fraction of each component. From the analysis of the mechanical properties, it can be concluded that the incorporation of oxidized carbon nano-onions increased the tensile strength of the material. This was because the amino groups of the CS and the carboxyl groups on the surface of the ox-CNO presented hydrogen bonds. Finally, the ox-CNO acted by absorbing part of the mechanical stress to which the films were exposed, improving their tensile strength [40].

##### Thermal Studies

The thermogravimetric analysis (TGA) (Figure S5, Supplementary Materials) showed the thermal behavior of the composites with three mayor decomposition stages. The first stage represented two degradation steps between 70 °C and 200 °C, due mainly to the water content of the films. The second degradation step started at 200 °C and ended at 390 °C due to CS and PVA decomposition. The final stage ranged between 409 °C for CS/PVA and 423 °C for CS/PVA/ox-CNO (29.00/70/1.0), which was attributed to the decomposition of the nano-onion content [83]. The ox-CNO incorporation into the composite CS/PVA increased the degradation temperatures (Table 2). The Td_3%_ (3% mass loss) values for all composites are exhibited in Table 2. The Td_3%_ increased for the composite with the lowest amount of ox-CNO (0.2 wt.%) due to the good dispersion in the polymer matrix generating hydrogen bonding between CS/PVA chains and ox-CNO, which suppressed the polymer chain mobility. However, as the ox-CNO loading further increased, the tendency for Td_3%_ was to decrease until F5, which had 1.0 wt.% OX-CNO, a high enough amount to once again increase the thermal and mechanical resistance. On the other hand, the reinforcement effect was also observed after the analysis of DSC. The glass transition temperature (T_g_) of F5 (ox-CNO 1.0 wt.%) increased from 78.4 to 78.7 °C, which also demonstrated a good dispersion and reinforcement of the CS/PVA composite (Table 2). However, no significant changes in T_g_ for the other formulations were observed as compared to the F1. This could also indicate a loss in crystallinity due to the low content of ox-CNO.

#### 3.2.5. Degradation in a Simulated Biological Fluid (FBS)

##### Weight Loss

**Figure 5:**
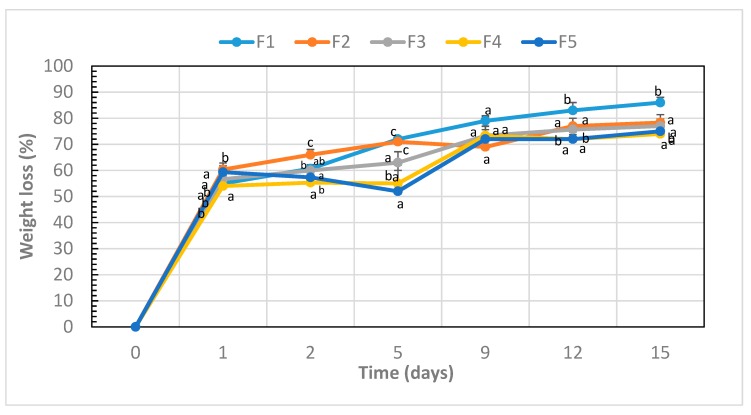
Weight loss vs. time of immersion for the films. Formulations: F1 (CS:PVA:ox-CNO 30:70:0); F2 (CS:PVA:ox-CNO 29.75:70:0.25); F3 (CS:PVA:ox-CNO 29.50:70:0.50); F4 (CS:PVA:ox-CNO 29.25:70:0.75); F5 (CS:PVA:ox-CNO 29.00:70:1.00). Different superscript letters indicate significant differences between treatments (*p* > 0.05).

Figure 5 shows the results of the degradation percentage (weight loss) of the films with different proportions of ox-CNO during 15 days of immersion in FBS. The incorporation of ox-CNO in the polymeric matrix increased the stability of the films in FBS since the percentage of weight loss after seven days of immersion decreased from 60% for F1 that did not contain ox-CNO to 53% for formulation F5 comprising 1.0% ox-CNO. This behavior was because the ox-CNO in the CS/PVA binary mixture gave the material a higher number of hydrogen bonds between the CS and the ox-CNO, which provided the material with high chemical stability in the polymer chains of the CS.

##### pH Changes

**Figure 6:**
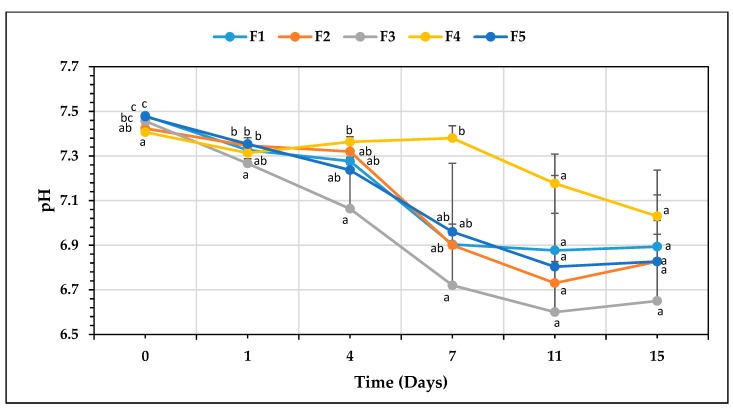
Study of the solution pH vs. immersion time in simulated biological fluid (FBS) of the films. Formulations: F1 (CS:PVA:ox-CNO 30:70:0); F2 (CS:PVA:ox-CNO 29.75:70:0.25); F3 (CS:PVA:ox-CNO 29.50:70:0.50); F4 (CS:PVA:ox-CNO 29.25:70:0.75); F5 (CS:PVA:ox-CNO 29.00:70:1.00). Different superscript letters indicate significant differences between treatments (*p* > 0.05).

Figure 6 shows the pH changes in the FBS during the immersion period of the samples. The decrease in pH value upon increasing the time of immersion in FBS was associated with the degradation of the amorphous zones which retained acidic species, as they have a greater susceptibility to degradation according to Figueira-Maldonado [96]. Furthermore, the detachment of the degradation by-products of CS such as glucosamine and *N*-acetylglucosamine contributed to the reduction in pH. These by-products of chitosan degradation are found in the extracellular matrix of human tissue [97]. The maintenance of biological pH media between 6 and 8 is fundamental for a cell’s enzymatic reactions [67,70,98]. All the formulations studied showed pH variations within the permissible range in the human body to promote life and maintenance of vital functions.

##### SEM of the Films after Immersion in FBS

**Figure 7:**
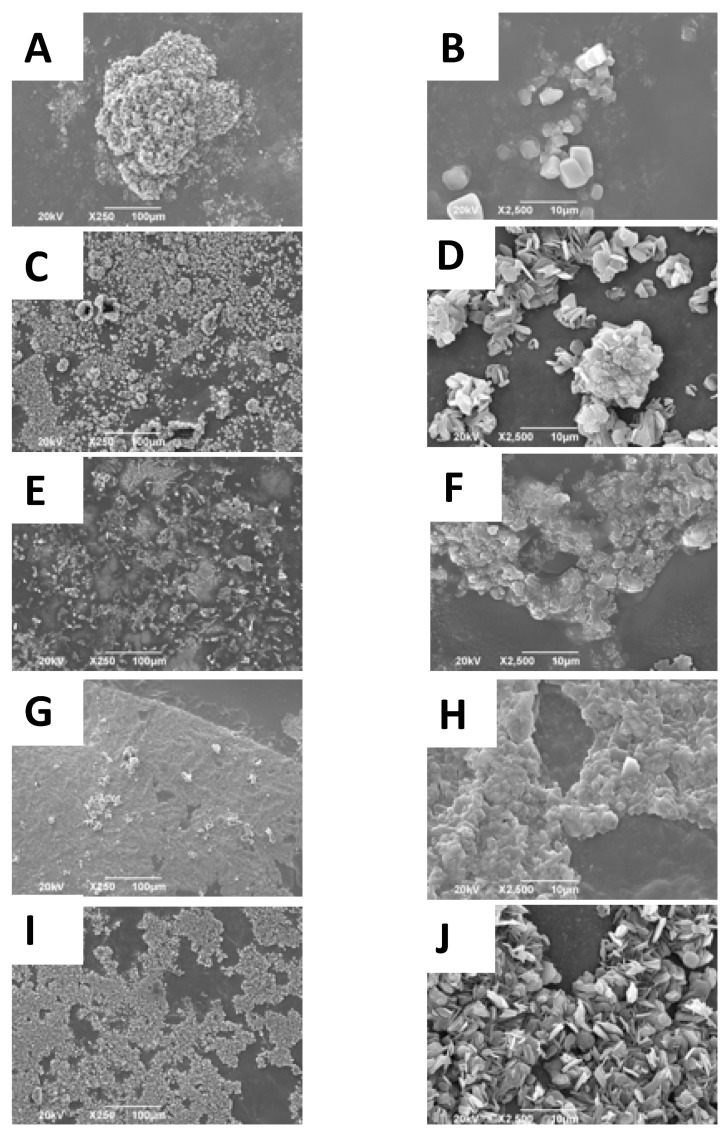
Morphology of CS/PVA/ox-CNO films after 15 days following the degradation process in FBS: F1 (30 CS/70 PVA/0 ox-CNO) (**A**) at 250×, (**B**) at 1000×; F2 (29.75 CS/70 PVA/0.25 ox-CNO) (**C**) at 250×, (**D**) at 1000×; F3 (29.50 CS/70 PVA/0.50 ox-CNO) (**E**) at 250×, (**F**) at 1000×; F4 (29.25 CS/70 PVA/0.25 ox-CNO) (**G**) at 250×, (**H**) at 1000×; F5 (29.00 CS/70 PVA/1.00 ox-CNO), (**I**) at 250× (**J**) at 1000×.

SEM images of the CS/PVA/ox-CNO films after their immersion in FBS are shown in Figure 7. The morphological structure of the deposited layer presented typical morphological characteristics of apatite structures (globular structures with several nucleation sites), similar to those reported by different authors for materials with bioactive traits [67]. The morphology of the films before and after the immersion in FBS were remarkably different as a product of the degradation process and the deposition of apatite-like salts. Roughness increased with the ox-CNO content, as well as the degradation process of the nanocomposite, which decreased with the ox-CNO content. A thin layer composed of calcium phosphate was also observed on the film’s surfaces, which could be determined by EDS with a calcium composition up to 12.74% and a phosphorus content of 1.21% for formulation F4 (Image S1, Supplementary Materials). This formulation had a high content of ox-CNO which demonstrates that the presence of the nanomaterial increased the affinity of the films for apatite layers. The deposition of apatite layers on the film’s surface could be a result of the biocompatibility of some materials with osteointegration ability [67]. However, to probe this statement, it was necessary to carry out in vivo studies with the material inside Wistar rats for 90 days.

#### 3.2.6. Biomodel Tests In Vivo

**Figure 8:**
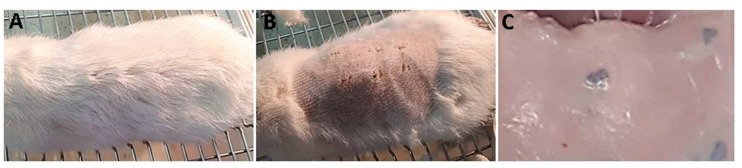
Zone of subdermal implantation in Wistar rats after 30, 60, and 90 days: (**A**) hair recovery, (**B**) absence of immune responses, and (**C**) subdermal implantation zones with samples encapsulated by scar tissue.

Nanocomposite films were recovered after 30, 60, and 90 days of implantation. In all cases, repair of the surgical defect and hair was observed (Figure 8A), even after 30 days of implantation, and the absence immune responses in the intervened areas (Figure 8B,C) demonstrated a normal biosorption process. The material was initially compatible, while an inflammatory reaction to a foreign body was apparent when the cells surrounded the fragments with a fibrous capsule, whereas the remaining soft tissue had a regular appearance.

**Figure 9:**
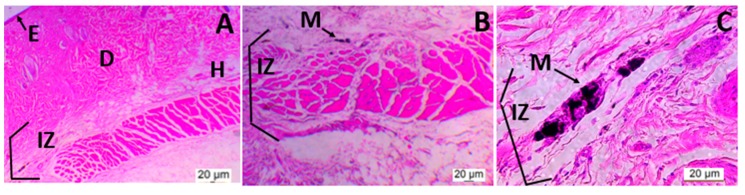
Histological analysis of experimental sample F1 (30 CS/70 PVA/0 ox-CNO). Magnifications are as follows: (**A**) 4×, (**B**) 10×, (**C**) 40×. E: Epidermis; D: Dermis; H: Hypodermis; IZ: Implantation zone; M: Material.

On the other hand, the results of the histological study demonstrated that the material was biocompatible and biodegradable even with a high content of ox-CNO. Figure 9 corresponds to the histological analysis of the subdermal tissues of biomodels (Wistar rats) implanted with films of the formulation F1 (30CS/70PVA/0 ox-CNO). After 60 days of implantation, most of the material (M) was reabsorbed (Figure 9A,B). Only a few small particles in the middle of an inflammatory infiltrate persisted (Figure 9C). At 90 days of implantation, the material was not appreciated either in macroscopic photographs or microscopic images.

Chitosan is a material that exhibits in vivo degradation associated with phagocytosis and enzymatic activity. The degradation rate depends on the degree of deacetylation, cross-linking, binding with other polymers, and forming scaffolds [99,100].

Several researchers demonstrated its biocompatibility, as well as its ability to degrade, inducing the regeneration of tissue. For example, Fujita et al. (2004) manufactured chitosan hydrogels that degraded after 20 days of subcutaneous implantation [101]. On the other hand, Vaishali et al. (2019) observed collagen sponges with some traces of chitosan remaining in subdermal sponges implanted in Wistar rats after 42 days, without adverse reactions of the immune system [102,103,104,105,106].

**Figure 10:**
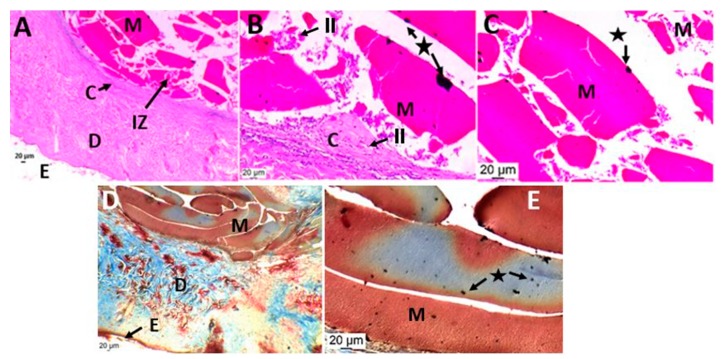
Histological analysis of experimental sample F2 (29.25 CS/70 PVA/0.25 ox-CNO). Magnifications are as follows: (**A**) 4×; (**B**) 10×; (**C**) 40×; (**D**) 4×; (**E**) 40×. E: Epidermis; D: Dermis; H: Hypodermis; C: Capsule; IZ: Implantation zone; M: Material; II: Inflammatory infiltrate. The stars correspond to places where ox-CNO particles were observed. (**A**–**C**) correspond to sample F2 stained using hematoxylin and eosin. (**D**,**E**) correspond to sample F2 stained using Masson’s trichromacy.

Here, the experimental formulation F2 (29.75CS/70PVA/0.25 ox-CNO) showed normal healing (Figure 10). However, after 60 and 90 days, large pieces of fragmented material persisted, and a small inflammatory infiltrate (II) could be observed in the capsule (C) and the implantation zone (IZ) (Figure 10B,C). However, the number of small pieces decreased after 30 more days of implantation (90 days of implantation), evidenced only inside the capsule (Figure 10D,E).

**Figure 11:**
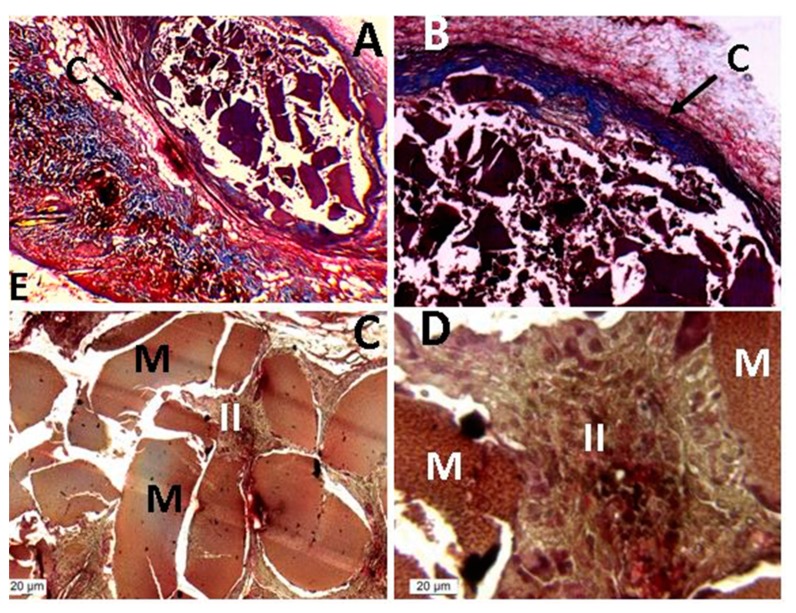
Histological analysis of experimental sample F3 (29.50 CS/70 PVA/0.50 ox-CNO. Magnifications are as follows: (**A**) 4×; (**B**) 10×; (**C**) 10×; (**D**) 40×. E: Epidermis; D: Dermis; H: Hypodermis; C: Capsule; IZ: Implantation zone; M: Material; II: Inflammatory infiltrate. (**A**,**B**) correspond to sample F3 stained using hematoxylin and eosin. (**C**,**D**) correspond to sample F2 stained using Masson’s trichromacy.

As with the F2 formulation, tissues implanted with F3 samples (29.50 CS/70 PVA/0.50 ox-CNO) presented a capsule after 30 days of implantation surrounding the implantation area, with some samples in the fragmentation process (Figure 11A,B). On the other hand, at 90 days of implantation, there was abundant inflammatory infiltrate (II) between the fragments of the material (M) (Figure 11).

**Figure 12:**
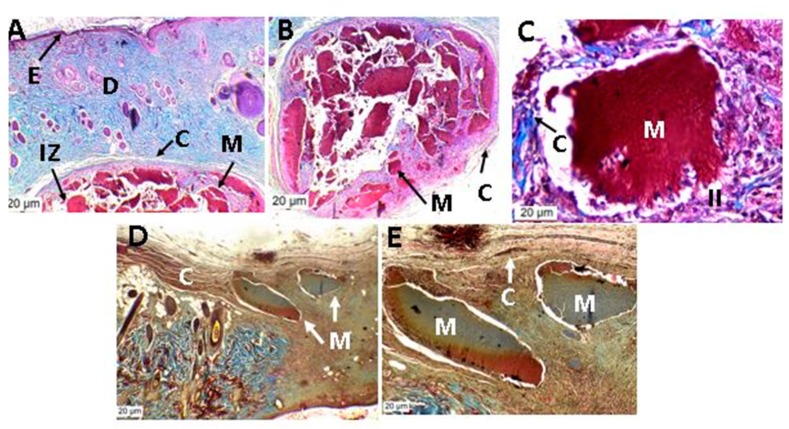
Histological analysis of experimental sample F4 (29.25 CS/70 PVA/0.75 ox-CNO). Magnifications are as follows: (**A**) 4×; (**B**) 10×; (**C**) 40×; (**D**) 4×; (**E**) 40×. E: Epidermis; D: Dermis; H: Hypodermis; C: Capsule; IZ: Implantation zone; M: Material; II: Inflammatory infiltrate. (**A**–**C**) correspond to sample F4 stained using hematoxylin and eosin. (**D**,**E**) correspond to sample F2 stained using Masson’s trichromacy.

The histological results (Figure 12) of samples of F4 (29.25 CS/70 PVA/0.75 ox-CNO) showed that, after 30 days, the implantation zone (IZ) was surrounded by a fibrous capsule (C) formed by type I collagen (Figure 12A,B), with a strong inflammatory activity inside that caused the reabsorption of the material (M).

Figure 12C corresponds to a magnification of the implantation zone at 40×, where it was observed that the fragment was also surrounded by a fibrous capsule, exhibiting phagocytic activity by inflammatory infiltrate cells (II).

Figure 12D,E correspond to samples of F4 after 60 days of implantation. The presence of the capsule, a lower activity of the inflammatory infiltrate, a highly fractionated material, and some spaces that correspond to already reabsorbed material are evident.

**Figure 13:**
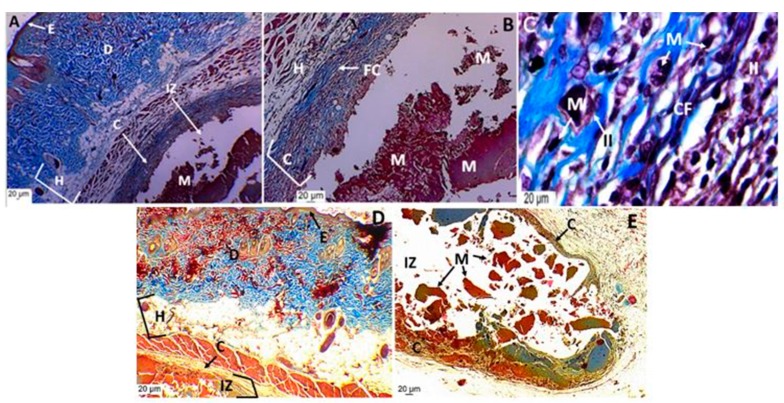
Histological analysis of experimental sample F5 (29.00 CS/70 PVA/1.0 ox-CNO). Magnifications are as follows: (**A**) 4×; (**B**) 10×; (**C**) 40×; (**D**) 4×; (**E**) 40×. E: Epidermis; D: Dermis; H: Hypodermis; C: Capsule; IZ: Implantation zone; M: Material; II: Inflammatory infiltrate. (**A**–**C**) correspond to sample F5 stained using hematoxylin and eosin. (**D**,**E**) correspond to sample F2 stained using Masson’s trichromacy.

The samples corresponding to the formulation F5 (Figure 13) at 30 days of implantation showed complete encapsulation, with fragmentation and partial degradation by phagocytosis, while, at 90 days, persistence of some fragments was observed without being reabsorbed.

The histological results exhibited a typical foreign body reaction caused by the films, which is the mechanism typically used by living beings to phagocytose subdermal implanted materials [63,64,65,66]. However, the introduction of higher amounts of ox-CNO seemed to stabilize the films (F3–F5) after 90 days of implantation. In the future, it will be necessary to measure the area of the remaining samples to determine the effectiveness of the degradation of the samples.

The sample without ox-CNO (F1) was the only one to show significant degradation, whereas the other formulations showed partial degradation at 60 and 90 days, demonstrating that ox-CNO acts by stabilizing the membrane and delaying its degradation. This behavior was also observed in the degradation with FBS, in thermal analysis, and in the analysis of mechanical properties. This could be a product of the filling effect caused by the interaction of ox-CNO with the PVA and CS chains, which stabilize the material and hinder the access of cells and macrophages for degradation.

## 4. Conclusions

The successful preparation of stable films based on CS/PVA/ox-CNO nanocomposites was confirmed from the mechanical, chemical, thermal, and biological analyses. The ox-CNO content influenced the stability of the films, which was confirmed by FBS hydrolytic degradation and histological studies. For the hydrolytic degradation, samples without ox-CNO content (F1) lost 80% of their weight after 15 days. This was correlated using in vivo studies following 90 days of subdermal implantation in Wistar rats, where no remaining material was found after 90 days. Upon the introduction of ox-CNO nanoparticles, the material seemed to stabilize due to chemical interactions with CS/PVA chains. Mechanical and thermal studies exhibited an increase in Young’s modulus and decomposition temperatures. DSC analysis showed that the nanofiller had a plasticizer effect, as the T_g_ decreased with ox-CNO content. In vivo studies showed some remaining material after 90 days of subdermal implantation in all the formulations with ox-CNO content, but without any immune response. However, this could be an advantage where long-term permanence is needed for applications, such as in skin loss due to trauma or burns, because the material was biocompatible, as confirmed by the results.

After FBS film hydrolytic degradation, a thin layer of calcium phosphate was deposited on the material surface, which is usually related to a bioactive material. Future work will include in vitro studies and critical size defect analysis to determine the tissue regeneration capacity of the CS/PVA/ox-CNO films.

## Figures and Tables

**Table 1:** Formulations used for the preparation of chitosan (CS)/poly(vinyl alcohol) (PVA)/oxidized carbon nano-onion (ox-CNO) nanocomposite solutions.

Component	F1	F2	F3	F4	F5
CS (%)	30	29.75	29.50	29.25	29
PVA (%)	70	70	70	70	70
ox-CNO (%)	0	0.25	0.50	0.75	1

**Table 2:** Td_3%_, Young’s modulus, and glass transition temperature (T_g_) of CS/PVA and CS/PVA/GO nanocomposites.

Sample *	ox-CNO (wt.%)	Td_3%_ (°C)	T_g_ (°C)	Young’s Modulus (GPa)
F1	0	67.3	78.4	3.73 ± 0.45
F2	0.25	75.0	77.1	2.80 ± 0.57
F3	0.50	50.8	78.2	3.20 ± 0.74
F4	0.75	71	78.0	3.51 ± 0.94
F5	1.00	85	78.7	3.86 ± 0.77

* Formulations: F1 (CS:PVA:ox-CNO 30:70:0); F2 (CS:PVA:ox-CNO 29.75:70:0.25); F3 (CS:PVA:ox-CNO 29.50:70:0.50); F4 (CS:PVA:ox-CNO 29.25:70:0.75); F5 (CS:PVA:ox-CNO 29.00:70:1.00).

## Supplementary Materials

The following are available online at https://www.mdpi.com/2218-273X/9/11/684/s1: Figure S1. Fourier-transform infrared spectroscopy (FTIR) of p-CNO (black line) and ox-CNO (red line); Figure S2. X-ray diffraction (DRX) of p-CNO (black line) and ox-CNO (red line); Figure S3. TGA of the oxidized carbon nano-onions and pristine carbon nano-onions; Figure S4. The Young’s modulus (left) and tensile strength (right) of CS/PVA/ox-CNO films; Formulations: F1 (CS:PVA:ox-CNO 30:70:0); F2 (CS:PVA:ox-CNO 29.75:70:0.25); F3 (CS:PVA:OX-CNO 29.50:70:0.50); F4 (CS:PVA:ox-CNO 29.25:70:0.75); F5 (CS:PVA:ox-CNO 29.00:70:1.00); Figure S5. TGA curves of the films. Formulations: F1 (CS:PVA:ox-CNO 30:70:0); F2 (CS:PVA:ox-CNO 29.75:70:0.25); F3 (CS:PVA:ox-CNO 29.50:70:0.50); F4 (CS:PVA:ox-CNO 29.25:70:0.75); F5 (CS:PVA:ox-CNO 29.00:70:1.00). Image S1. Energy-dispersive X-ray spectroscopy (EDS) results of the formulations F1–F5.
