# Supplementary material for: Preparation of Chitosan/Poly(Vinyl Alcohol) Nanocomposite Films Incorporated with Oxidized Carbon Nano-Onions (Multi-Layer Fullerenes) for Tissue-Engineering Applications
Source: Biomolecules. 2019 Nov 1;9(11):684. doi: 10.3390/biom9110684 (PMC6920947; doi:10.3390/biom9110684)
Supplement: Supplementary file 1 [file biomolecules-09-00684-s001.pdf]

Supporting file

Preparation of chitosan/Poly (vinyl alcohol) nanocomposite films incorporated with oxidized carbon nano-onions (multi-layer fullerenes) for tissue engineering applications

Carlos David Grande Tovar[1], Jorge Iván Castro[2], Carlos Humberto Valencia[3], Diana Paola Navia Porras[4], José Herminsul Mina Hernandez[5]*, Mayra Eliana Valencia[5], José Daniel Velásquez[2], and Manuel Noé Chaur[2,6]*

[1] Grupo de investigación de fotoquímica y fotobiología, Universidad del Atlántico, Carrera 30 Número 8-49, Puerto Colombia 081008, Colombia; carlosgrande@mail.uniatlantico.edu.co (C.D.G.T.)

[2] Grupo de Investigación SIMERQO, Departamento de Química, Universidad del Valle, Calle 13 No. 100-00, 76001 Cali, Colombia; jorgecastro@correounivalle.edu.co; manuel.chaur@correounivalle.edu.co (M.N.C.) (J.I.C.); jose.velasquez.carmona@correounivalle.edu.co (J.D.V.)

[3] Escuela de Odontología, Grupo biomateriales dentales, Universidad del Valle, Calle 13 No. 100-00, 76001 Cali, Colombia; carlos.humberto.valencia@correounivalle.edu.co (C.H.V.)

[4] Grupo de Investigación Biotecnología, Facultad de Ingeniería, Universidad de San Buenaventura Cali, Carrera 122 # 6-65, 76001 Cali, Colombia; dpnavia@usbcali.edu.co (D.P.N.P.)

[5] Escuela de Ingeniería de Materiales, Facultad de Ingeniería, Universidad del Valle, Calle 13 No. 100-00, Santiago de Cali 760032, Colombia; valencia.mayra@correounivalle.edu.co (M.E.V.); jose.mina@correounivalle.edu.co (J.H.M.)

[6] Materiales (CENM), Universidad del Valle, Calle 13 No. 100-00, Santiago de Cali 760032, Colombia; manuel.chaur@correounivalle.edu.co (M.N.C.)

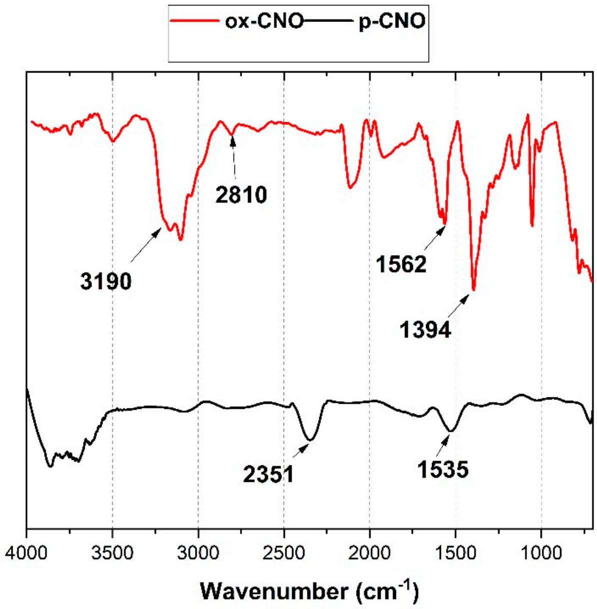

Figure S1. Fourier transform infrared spectroscopy (FTIR) of p-CNO (black line) and ox-CNO (red line).

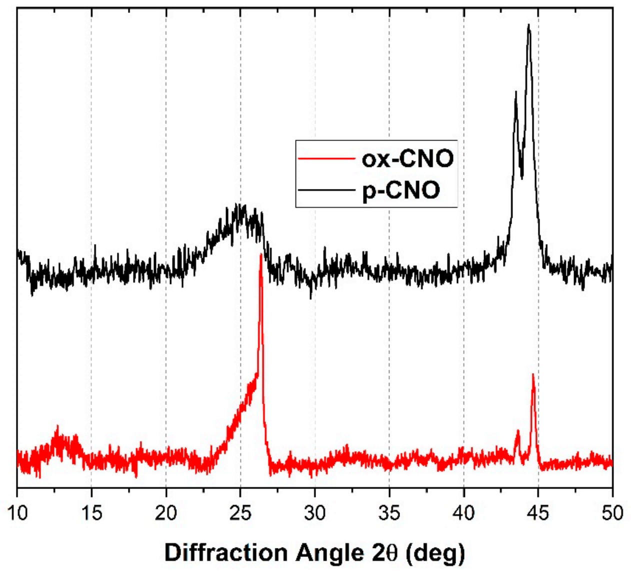

Figure S2. X-ray diffraction (DRX) of p-CNO (black line) and OX-CNO (red line).

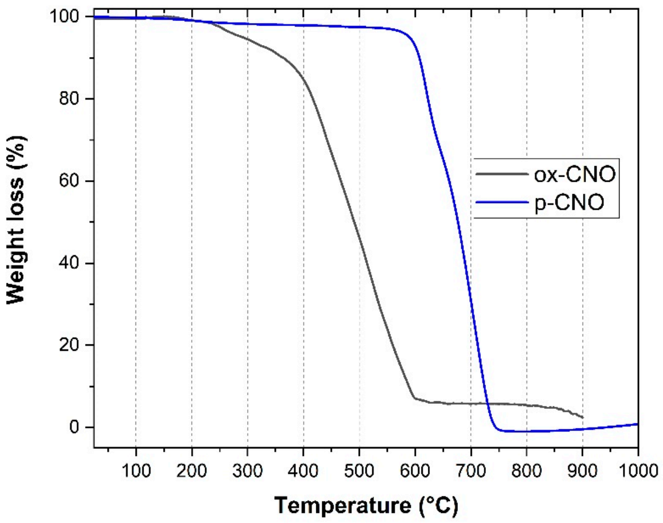

Figure S3. TGA of the oxidized carbon nano-onions and pristine carbon nano-onions.

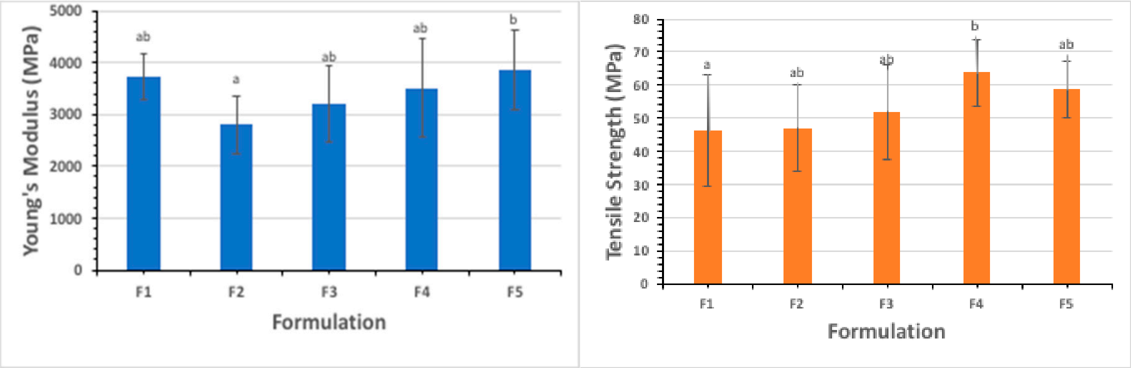

Figure S4. The Young's modulus (left) and tensile strength (right) of CS/PVA/ox-CNO films. Formulations: F1 (CS:PVA:ox-CNO 30:70:0); F2 (CS:PVA:ox-CNO 29.75:70:0.25); F3 (CS:PVA:OX-CNO 29.50:70:0.50); F4 (CS:PVA:ox-CNO 29.25:70:0.75);F5 (CS:PVA:ox-CNO 29.00:70:1.00). Different superscript letters indicate significant differences between treatments ($p > 0.05$).

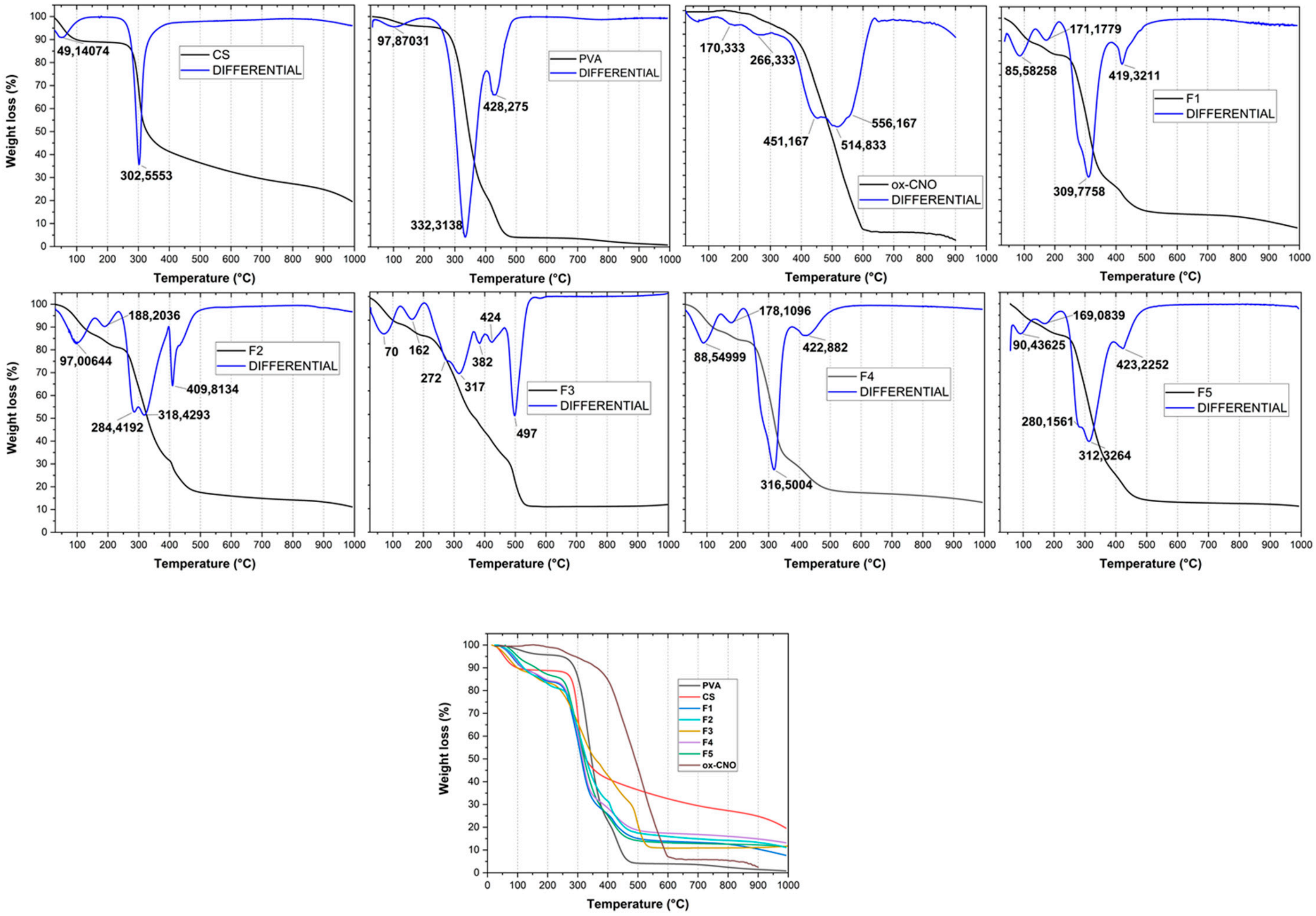

Figure S5. TGA curves of the films. Formulations: F1 (CS:PVA:ox-CNO 30:70:0); F2 (CS:PVA:ox-CNO 29.75:70:0.25); F3 (CS:PVA:ox-CNO 29.50:70:0.50); F4 (CS:PVA:ox-CNO 29.25:70:0.75);F5 (CS:PVA:ox-CNO 29.00:70:1.00)

- **EDS images for all the formulations (F1-F5)**

F1

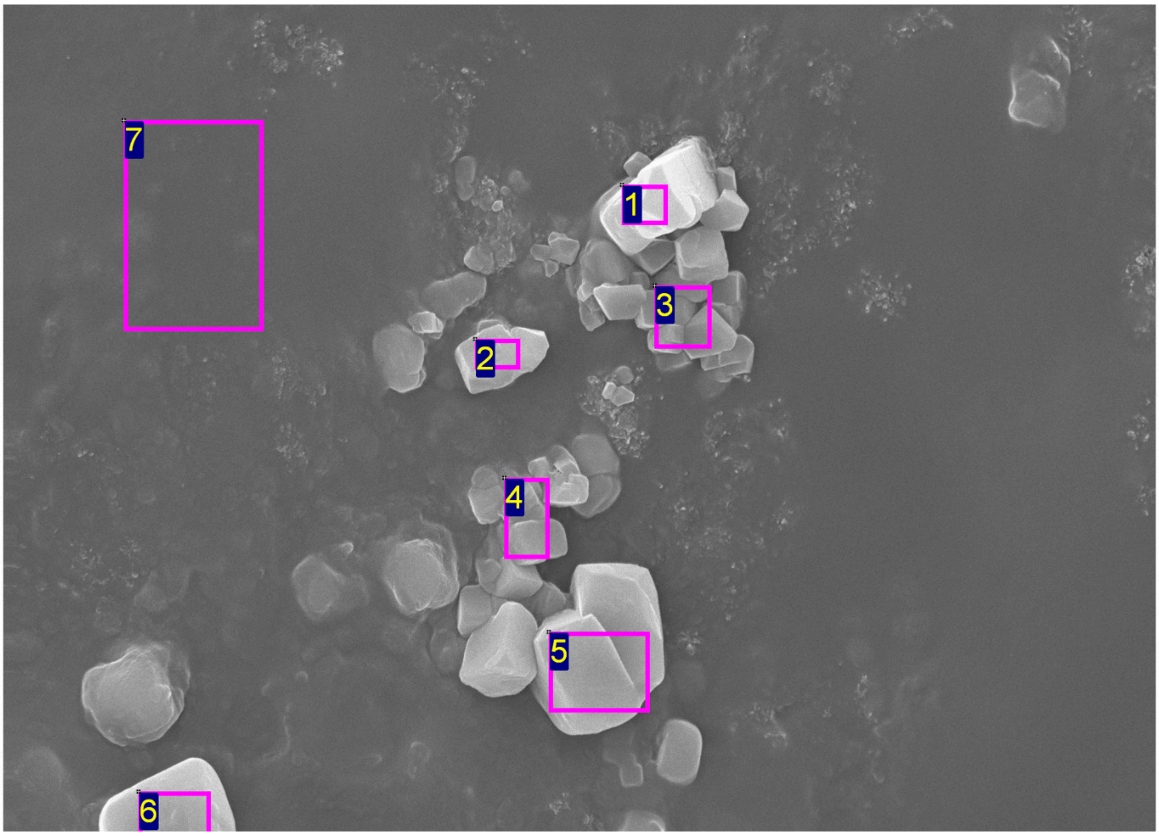

Processing option : All elements analysed (Normalised)

Spectrum	In stats.	B	C	O	Cl	K	Ca	Total
Spectrum 1	Yes		35.26		31.22	33.52		100.00
Spectrum 2	Yes		35.95		30.89	33.16		100.00
Spectrum 3	Yes		17.02		39.78	43.20		100.00
Spectrum 4	Yes			13.46	41.82	44.72		100.00
Spectrum 5	Yes	59.74	5.28		17.50	17.48		100.00
Spectrum 6	Yes		25.64		35.81	38.55		100.00
Spectrum 7	Yes		43.73	29.22	16.68	4.85	5.52	100.00
Max.		59.74	43.73	29.22	41.82	44.72	5.52	
Min.		59.74	5.28	13.46	16.68	4.85	5.52	

All results in weight%

F2

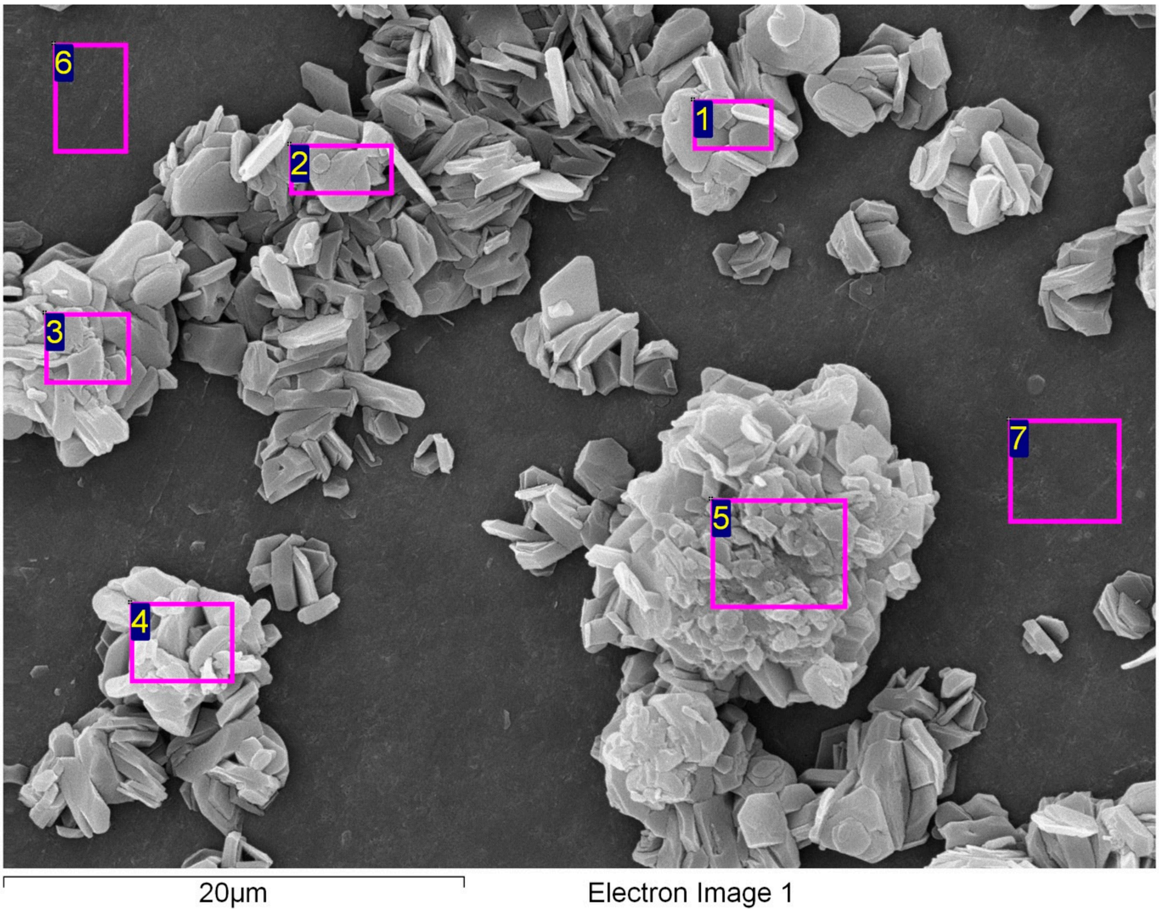

Processing option : All elements analysed (Normalised)

Spectrum	In stats.	C	N	O	Na	P	Cl	K	Ca	Total
Spectrum 1	Yes	44.90		34.09			17.38		3.63	100.00
Spectrum 2	Yes	44.82		33.83			19.07		2.27	100.00
Spectrum 3	Yes			56.40			39.43		4.17	100.00
Spectrum 4	Yes		21.50	50.66	2.09		21.47		4.28	100.00
Spectrum 5	Yes			50.24			49.76			100.00
Spectrum 6	Yes			53.81			35.65		10.54	100.00
Spectrum 7	Yes	30.37		32.30	3.35	1.21	16.12	2.34	14.31	100.00
Max.		44.90	21.50	56.40	3.35	1.21	49.76	2.34	14.31	
Min.		30.37	21.50	32.30	2.09	1.21	16.12	2.34	2.27	

All results in weight%

F3

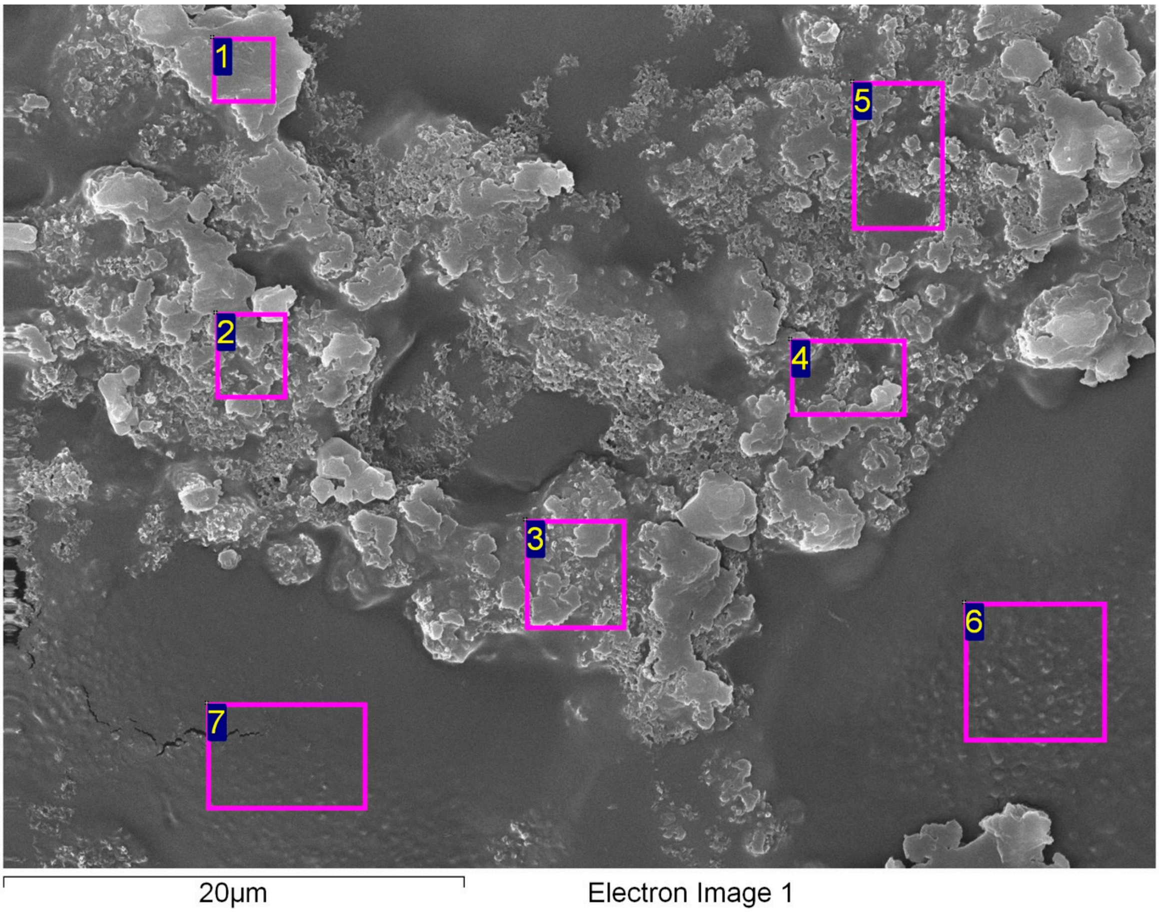

Processing option : All elements analysed (Normalised)

Spectrum	In stats.	C	O	Na	P	Cl	K	Ca	Total
Spectrum 1	Yes		41.36	4.24		25.35	16.31	12.74	100.00
Spectrum 2	Yes		33.27	3.40	-0.51	29.27	23.66	10.91	100.00
Spectrum 3	Yes		32.39	2.33	1.91	28.70	25.84	8.84	100.00
Spectrum 4	Yes		47.17	2.73	0.92	23.03	15.14	11.01	100.00
Spectrum 5	Yes	41.77	32.91	1.78	1.34	9.86	4.15	8.18	100.00
Spectrum 6	Yes	41.60	39.13			12.11	1.79	5.37	100.00
Spectrum 7	Yes	46.46	34.53			13.61		5.41	100.00
Max.		46.46	47.17	4.24	1.91	29.27	25.84	12.74	
Min.		41.60	32.39	1.78	-0.51	9.86	1.79	5.37	

All results in weight%

F4

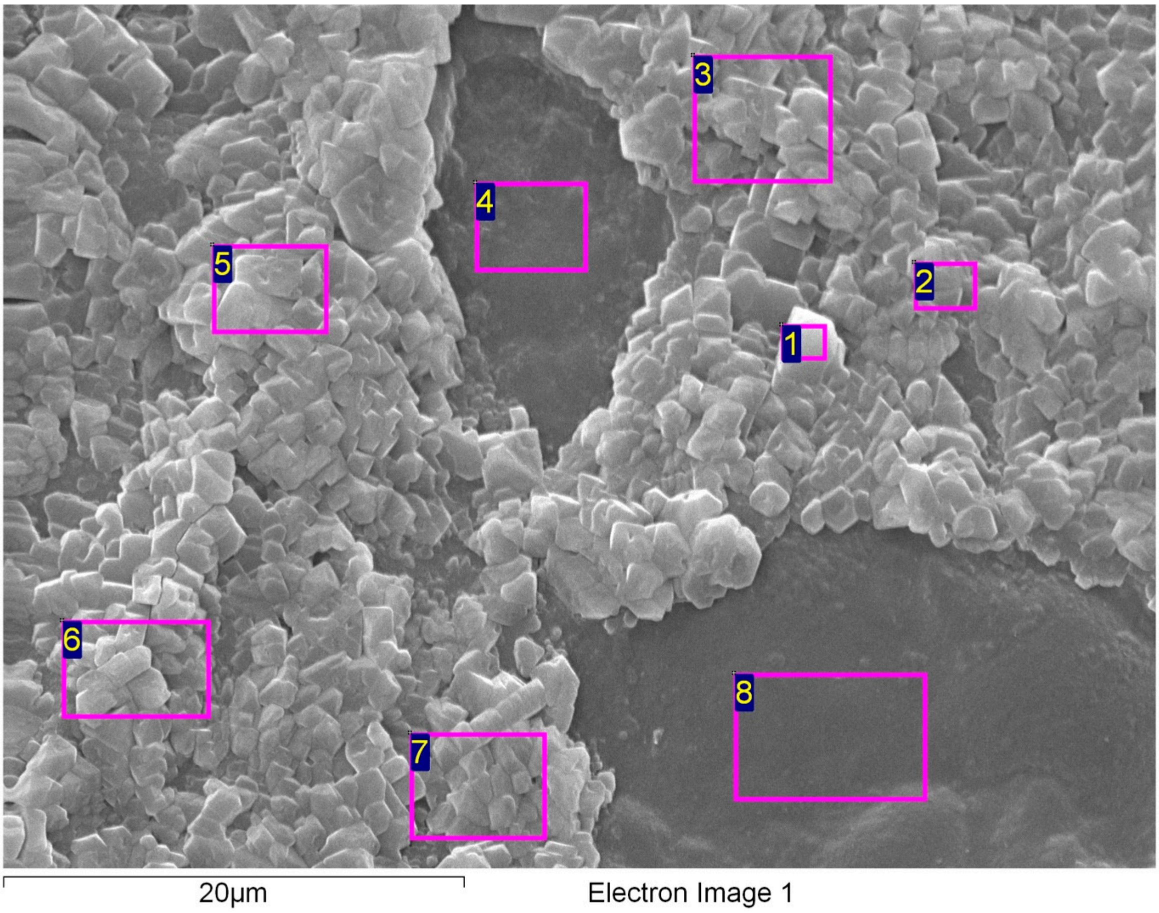

Processing option : All elements analysed (Normalised)

Spectrum	In stats.	C	O	Na	Cl	Ca	Total
Spectrum 1	Yes	31.70		27.72	40.59		100.00
Spectrum 2	Yes			34.53	65.47		100.00
Spectrum 3	Yes			35.29	64.71		100.00
Spectrum 4	Yes		17.25	27.02	54.31	1.43	100.00
Spectrum 5	Yes			37.44	62.56		100.00
Spectrum 6	Yes			35.88	64.12		100.00
Spectrum 7	Yes	26.39		26.68	46.93		100.00
Spectrum 8	Yes		19.11	24.79	56.10		100.00
Max.		31.70	19.11	37.44	65.47	1.43	
Min.		26.39	17.25	24.79	40.59	1.43	

All results in weight%

F5

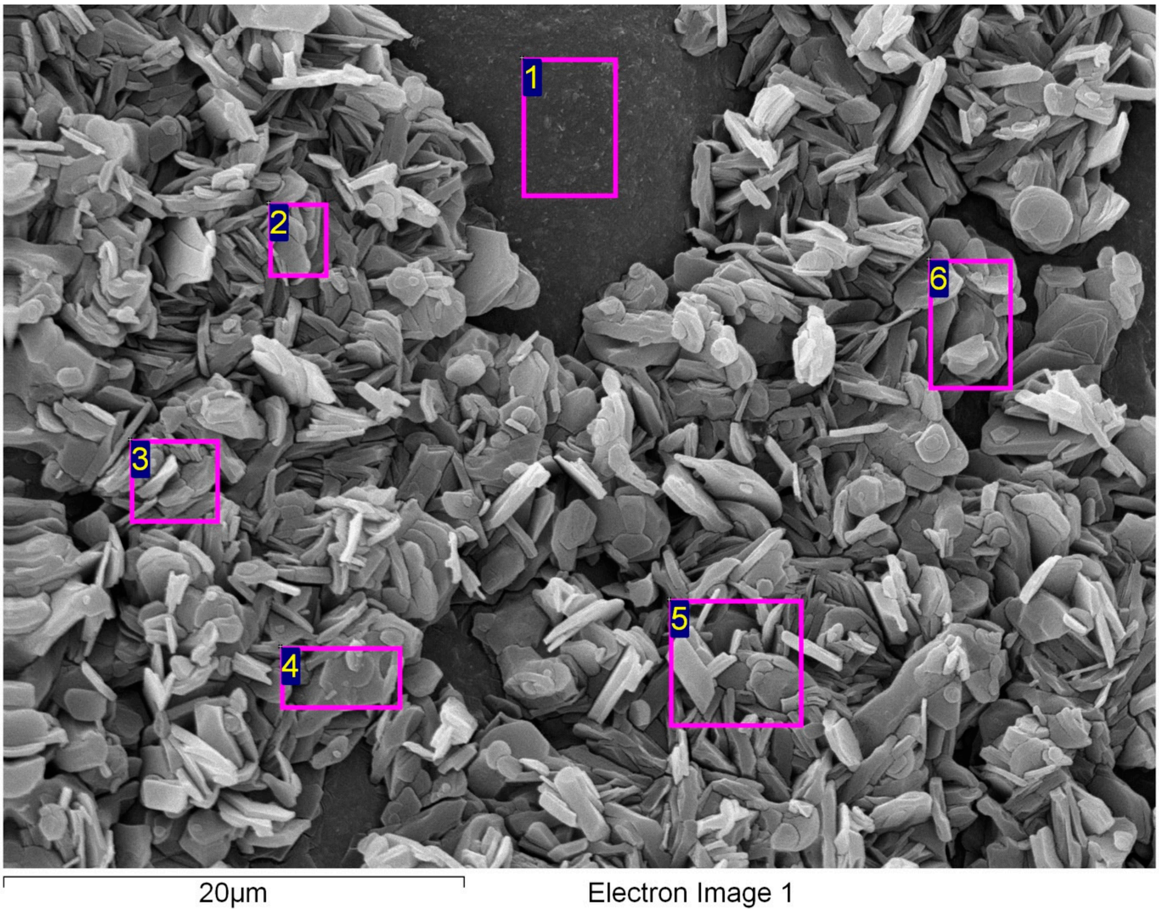

Processing option : All elements analysed (Normalised)

Spectrum	In stats.	C	O	Cl	Ca	Total
Spectrum 1	Yes	41.65	39.52	13.75	5.08	100.00
Spectrum 2	Yes		48.63	51.37		100.00
Spectrum 3	Yes		53.92	46.08		100.00
Spectrum 4	Yes		49.56	47.06	3.38	100.00
Spectrum 5	Yes		53.65	44.09	2.27	100.00
Spectrum 6	Yes		55.22	39.68	5.11	100.00
Max.		41.65	55.22	51.37	5.11	
Min.		41.65	39.52	13.75	2.27	

All results in weight%

Image S1. Energy-dispersive X-ray spectroscopy (EDS) results of the formulations F1-F5.
